# Inflammatory Profiles Induced by Intranasal Immunization with Ricin Toxin-immune Complexes

**DOI:** 10.4049/immunohorizons.2400007

**Published:** 2024-06-26

**Authors:** Lindsey E. Tolman, Nicholas J. Mantis

**Affiliations:** * Department of Biomedical Sciences, School of Public Health, University at Albany, Albany, NY; † Division of Infectious Diseases, Wadsworth Center, New York State Department of Health, Albany, NY

## Abstract

The underlying contribution of immune complexes in modulating adaptive immunity in mucosal tissues remains poorly understood. In this report, we examined, in mice, the proinflammatory response elicited by intranasal delivery of the biothreat agent ricin toxin (RT) in association with two toxin-neutralizing mAbs, SylH3 and PB10. We previously demonstrated that ricin-immune complexes (RICs) induce the rapid onset of high-titer toxin-neutralizing Abs that persist for months. We now demonstrate that such responses are dependent on CD4^+^ T cell help, because treatment of mice with an anti-CD4 mAb abrogated the onset of RT-specific Abs following intranasal RICs exposure. To define the inflammatory environment associated with RIC exposure, we collected bronchoalveolar lavage fluid (BALF) and sera from mice 6, 12, and 18 h after they had received RT or RICs by the intranasal route. A 32-plex cytometric bead array revealed an inflammatory profile elicited by RT that was dominated by IL-6 (>1500-fold increase in BALF) and secondarily by KC (CXCL1), G-CSF, GM-CSF, and MCP-1. RICs induced inflammatory profiles in both BALF and serum response that were similar to RT, albeit at markedly reduced levels. These results demonstrate that RICs retain the capacity to induce local and systemic inflammatory cytokines/chemokines that, in turn, may influence Ag sampling and presentation in the lung mucosa and draining lymph nodes. A better understanding of the fate of immune complexes following intranasal delivery has implications for the development of mucosal vaccines for biothreats and emerging infectious diseases.

## Introduction

Inhalation of the biothreat agent ricin toxin (RT) provokes a localized inflammatory response that involves pulmonary congestion, edema, neutrophil infiltration, and severe acute respiratory distress (1). RT’s binding subunit (RTB), a galactose/N-acetyl galactosamine (Gal/GalNAc)-specific lectin, mediates toxin uptake into airway epithelial cells and other cell types within the mucosa (2, 3). Within the airway, RT is internalized by alveolar macrophages via mannose receptor–mediated recognition of RTB’s two-mannose side chain (4). RT’s enzymatic subunit (RTA) is a ribosome-inactivating protein that triggers cellular stress and programmed cell death pathways, resulting in the release of high levels of proinflammatory cytokines and chemokines (5–7).

Although there are currently no Food and Drug Administration–approved medical countermeasures for pulmonary RT exposure, early intervention with the toxin-neutralizing mAb PB10, which targets an immunodominant epitope on RTA, rescued Rhesus macaques from an otherwise lethal dose of aerosolized toxin (8). The same mAb given to nonhuman primates in advance of RT exposure conferred near-complete protection against toxin-induced morbidity and mortality (9). The efficacy of PB10 is further improved when combined with a second toxin-neutralizing mAb, SylH3, directed against RTB (10–12). Interestingly, although the PB10/SylH3 mixture virtually eliminates RT-induced pulmonary toxicity and tissue damage, the Ab mixture only marginally reduces RT binding to target cells in the lung tissues (11). Therefore, we have postulated that PB10 and SylH3 neutralize RT by interfering with RTB- and MR-mediated uptake and/or intracellular trafficking rather than blocking attachment to target cells (11, 13–15).

We were intrigued by the possibility that therapeutic Abs such as PB10 and SylH3 might influence the immunological fate of RT, because immune complexes (ICs) are known to have immunomodulatory activity (16). Indeed, we found that intranasal (i.n.) administration of ricin immune complexes (RICs) consisting of RT and equimolar amounts of PB10 and SylH3 resulted in the rapid onset of anti-RT IgG Abs that persisted for months (17). Moreover, a single i.n. dose of RIC was sufficient to confer protection against RT challenge 30 or 90 d later. The induction of RT-specific serum IgG was independent of FcγR engagement, as revealed through FcγR knockout mice and PB10/SylH3 LALA (leucine to alanine) derivatives. These observations suggested that RT as a preformed IC has the potential to generate a humoral response akin to vaccination. Understanding this phenomenon could potentially open up new avenues for both RT vaccine development and the expansion of therapeutic mAb applications for preventive purposes. In the present study, we set out to define the role of Th cells and characterize the local and systemic inflammatory environments in the context of i.n. RICs immunization.

## Materials and Methods

### Chemicals, biological reagents, and mAbs

RT (*Ricinus communis* agglutinin II; RCA_60_) was custom ordered from Vector Laboratories (Burlingame, CA) and dialyzed against PBS at 4°C in 10,000 MWCO Slide-A-Lyzer dialysis cassettes (Thermo Fisher Scientific, Pittsburgh, PA) prior to use in mice. Murine mAbs PB10 and SylH3 were purified by protein G chromatography by the Dana-Farber Cancer Institute’s Monoclonal Antibody Core Facility. PB10 is a murine IgG2b that targets RTA and SylH3 an IgG1 targeting RTB (18–21). Unless noted otherwise, all reagents were purchased from Sigma-Aldrich (St. Louis, MO).

### Mouse studies

The mouse studies were conducted under strict compliance with the Wadsworth Center’s Institutional Animal Care and Use Committee. Female BALB/c mice aged 7–8 wk were purchased from Taconic Biosciences (Rensselaer, NY). For RIC immunizations, RT (1 μg) was mixed with 20 μg each of PB10 IgG2b and SylH3 IgG1 and administered to mice in a final volume of 40 μl i.n. For RT-only exposures, 5 × LD_50_ (1 μg unless otherwise noted) was administered to mice in a volume of 40 μl for i.n. delivery. All i.n. inoculations were performed on isoflurane-anesthetized animals, which were monitored after the procedure to ensure normal recovery from anesthesia. Following RT exposure, mice were monitored daily for 7 d for symptoms of ricin intoxication, including weight loss, low blood glucose levels, and clinical signs of morbidity. Morbidity was measured using an institutional animal care and use committee–approved grading sheet stratified on a scale from 0 to 3, with 0 indicating normal activity and appearance and 3 indicating severe illness.

Mice were euthanized by CO_2_ asphyxiation followed by cervical dislocation when they exceeded predetermined thresholds for weight loss, blood glucose levels, or physical signs of morbidity. For studies requiring harvest of bronchoalveolar lavage fluid (BALF), mice were euthanized by CO_2_ asphyxiation and subsequent exsanguination via the abdominal aorta. For collection of BALF, 1 ml 0.6 mM EDTA in PBS was injected into the lungs intratracheally and immediately recollected. BALF samples were stored at −80°C until use. Blood was procured from the submandibular vein and serum isolated as described (17).

### CD4^+^ T cell depletion

Mice were i.p. injected with 200 μg of rat anti-mouse CD4 mAb (clone GK1.5; Thermo Fisher Scientific) 1 d prior to RIC immunization. The same dose and delivery route were used for subsequent treatment on days 1 and 4 following RIC immunization. For confirmation of Ab efficacy, mice were i.p. injected with 200 μg rat anti-mouse CD4 mAb 1 d before harvest of splenocytes for analysis of CD4^+^ populations by flow cytometry in comparison with splenocytes from untreated animals. The following Abs were used for labeling cells for flow cytometry: PE rat anti-mouse CD45 (clone 30-F11; BioLegend), FITC rat anti-mouse CD3 (clone 17A2; BD Biosciences, San Jose, CA), and allophycocyanin rat anti-mouse CD4 (clone RM4-5; BD Biosciences).

### IL-6 depletion

Mice were i.p. injected with 400 μg *InVivo*MAb anti-mouse IL-6 mAb (clone MP5-20F3; Bio X Cell, Lebanon, NH) 1 d prior to RIC immunization. The same dose and delivery route were used for treatment 1 d after RIC immunization. For confirmation of Ab efficacy, mice were i.p. injected with 400 μg *InVivo*MAb anti-mouse IL-6 mAb 1 d before RIC immunization. On the day following immunization, BALF was collected for analysis of secreted IL-6 by Luminex in comparison with BALF from immunized animals that did not receive anti-mouse IL-6 treatment.

### ELISA

Ninety-six-well high-binding microtiter plates (Thermo Fisher Scientific) were coated overnight with RT (0.1 μg/well in PBS) at 4°C. The plates were then washed and blocked as described (17) before the addition of mouse sera or mAbs. Sera and mAbs were diluted in ELISA block solution (5% goat serum, 0.1% Tween 20 in PBS). HRP-labeled goat anti-mouse IgG polyclonal Abs (SouthernBiotech, Birmingham, AL) were diluted 1:2000 in block solution prior to use. Plates were developed using 3,30,5,50-tetramethylbenzidine (Kirkegaard & Perry Labs, Gaithersburg, MD) and analyzed with a SpectraMax iD3 spectrophotometer equipped with SoftMax Pro 7.1 software (Molecular Devices, Sunnyvale, CA).

### Multiplex immunoassays

The MILLIPLEX Mouse Cytokine/Chemokine Magnetic Bead Panel (MCYTMAG-70K-PX32) from MilliporeSigma (Burlington, MA) was used to quantify cytokine contents from serum and BALF samples according to the manufacturer’s instructions. Serum and BALF samples were diluted in an equal volume of assay buffer and PBS, respectively, then added to premixed beads in a 96-well microtiter plate for overnight incubation at 4°C with shaking. Plates were washed using a handheld plate magnet before addition of primary Abs followed by incubation at room temperature for 1 h with shaking. Streptavidin-PE was added to each well and incubated with shaking for 30 min at room temperature. Plates were washed as described above and run in Sheath Fluid PLUS on a Luminex FLEXMAP 3D with INTELLIFLEX software (Luminex Corp., Austin, TX). Values were recorded as median fluorescence intensity. For analysis, assay kit standards were used to interpolate median fluorescence intensity values of each cytokine in each sample into a pg/ml value per the manufacturer’s instructions before adjusting for dilution factors and normalizing to background.

### Flow cytometry

To prepare splenocyte samples, harvested spleens were collected in 10 ml HBSS on ice before manual digestion through a 70-μm nylon mesh strainer. Cells were centrifuged and resuspended in lysis buffer (150 mM ammonium chloride [NH_4_Cl], 0.7 mM potassium phosphate monobasic [KH_2_PO_4_] in dH_2_O) for 4 min at room temperature with agitation. HBSS was added to samples before centrifugation, counting, and resuspension at 5 × 10^6^ cells/ml in ice-cold FACS buffer (1% FBS, 1 mM EDTA in PBS). For all experiments, 500 μl cell suspension was added to 12 × 75–mm polystyrene tubes and incubated with 100 μl TruStain FcX (BioLegend, San Diego, CA) on ice for 20 min. The following Abs were used for labeling cells: PE rat anti-mouse CD45 (clone 30-F11; BioLegend), FITC rat anti-mouse CD3 (clone 17A2; BD Biosciences, San Jose, CA), and allophycocyanin rat anti-mouse CD4 (clone RM4-5; BD Biosciences). A quantity of 50 μl of each Ab was added to samples and incubated on ice in the dark for 1 h. Cells were fixed using BD Cytofix Fixation Buffer (BD Biosciences) according to the manufacturer’s instructions and stored at 4°C protected from light before acquisition on a FACSCalibur cytometer equipped with CellQuest Pro software (BD Biosciences) within 1 wk of fixation. Analysis was performed using FlowJo version 10.8.0 (BD Biosciences).

### Statistical analyses

Statistical analyses of weights and titers were carried out using GraphPad Prism version 9.2.0. Unpaired, two-tailed Welch’s *t* tests were performed to determine the significance of differences in endpoint titers between groups, as well as in cytokine contents in BALF and serum. In all cases, *p ≤ 0.05, **p ≤ 0.01, ***p ≤ 0.001, and ****p < 0.0001.

## Results

### Role of CD4^+^ T cells in stimulating de novo RT-specific IgG following intranasal RIC immunization

**FIGURE 1.**
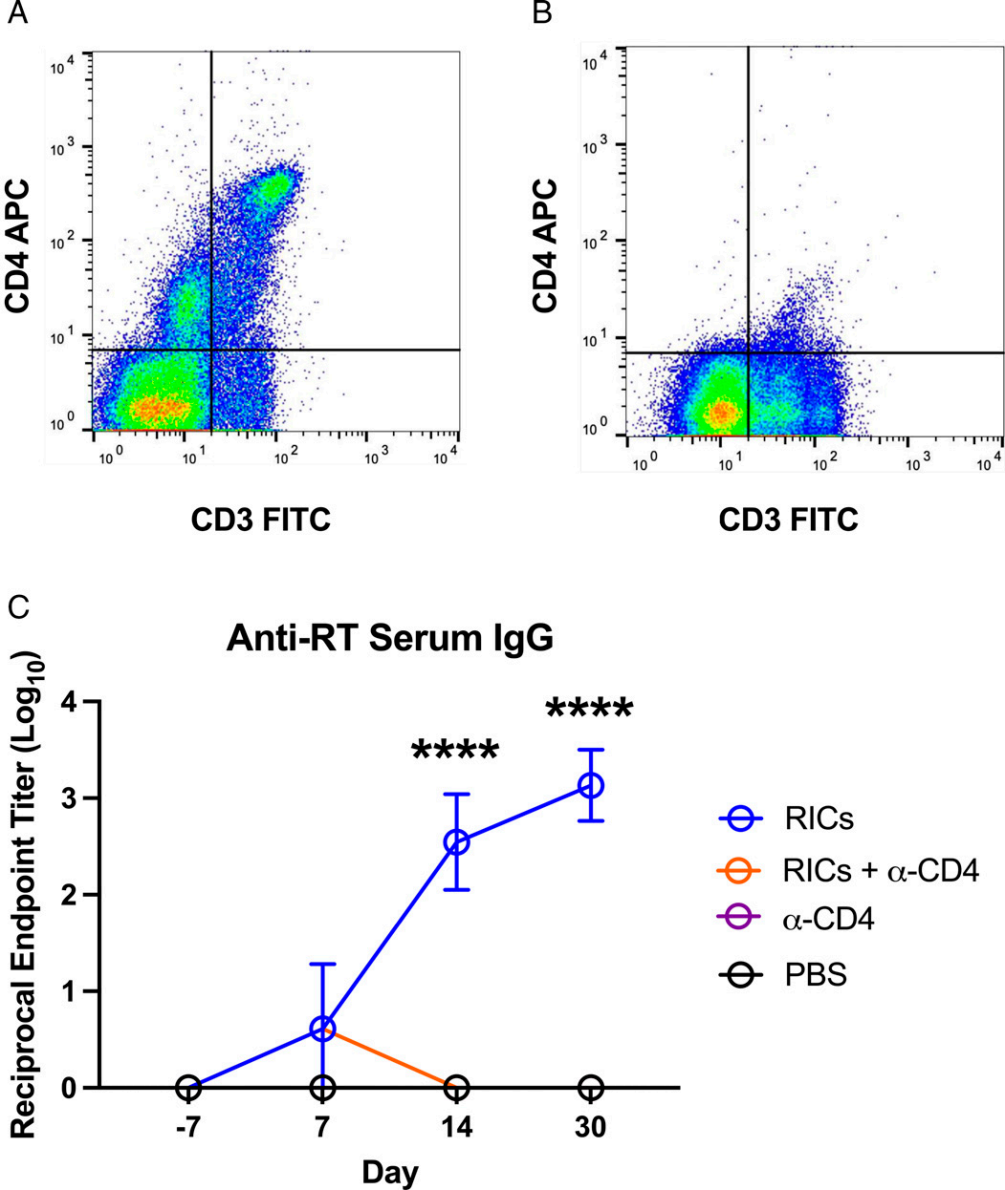
Role of CD4^+^ T cells in mounting a humoral response following i.n. RIC immunization. RICs were administered i.n. alone or in tandem with anti-CD4 treatment. (**A**) Combined population of Th cells (CD3^+^CD4^+^) in the spleens of untreated mice (*n* = 3). (**B**) Combined population of Th cells in the spleens of mice treated with anti-CD4 Ab 24 h prior to harvest (*n* = 3). (**C**) Anti-RT serum IgG titers of RIC-immunized mice receiving no additional treatment or anti-CD4 Ab (*n* = 6) or control animals receiving only anti-CD4 Ab or PBS (*n* = 4). Anti-CD4 treatment mice received anti-CD4 mAb 24 h prior to RIC vaccination, as well as 1 and 4 d postimmunization to ensure continual depletion. Day −7 is shown as a negative control time point prior to any RIC or anti-CD4 treatments. Significance of endpoint titers was determined by unpaired, two-tailed Welch’s *t* test. ****p < 0.0001.

We recently reported that i.n. administration of RICs to mice induces a rapid and durable serum anti-RT IgG response within 10–14 d without a detectable IgM phase. To ascertain whether CD4^+^ T cells are involved in this response, groups of adult BALB/c mice were treated with an anti-CD4 mAb (400 µg by i.p. injection) before (d −1) and after (d +1, +4) i.n. RIC administration on study day 0. The anti-CD4 Ab regimen resulted in a near-complete of depletion of CD4^+^ T cells, as determined in a parallel group of mice (Fig. 1A, 1B). RT-specific serum IgG titers were measured in sera collected on days 7, 14, and 30 following RIC administration. In the control group of mice, i.n. RIC administration resulted in the onset of RT-specific serum IgG by day 14, with reciprocal endpoint titers exceeding 10^3^ by day 30 (Fig. 1C). Although IgG subclass profiles were not determined in this study, we previously demonstrated that RIC vaccination elicits an IgG1-dominated response (17). By comparison, in mice treated with the anti-CD4 mAb, RT-specific IgG levels were at baseline, even on day 30 (Fig. 1C). This result demonstrates the necessity of CD4^+^ T cells in the humoral response to RICs and specifically warrants an investigation into the role of T follicular helper cells in this process, especially in the draining lymph nodes (22).

### Induction of mucosal and systemic inflammatory cytokines and chemokines following RIC exposure

**FIGURE 2.**
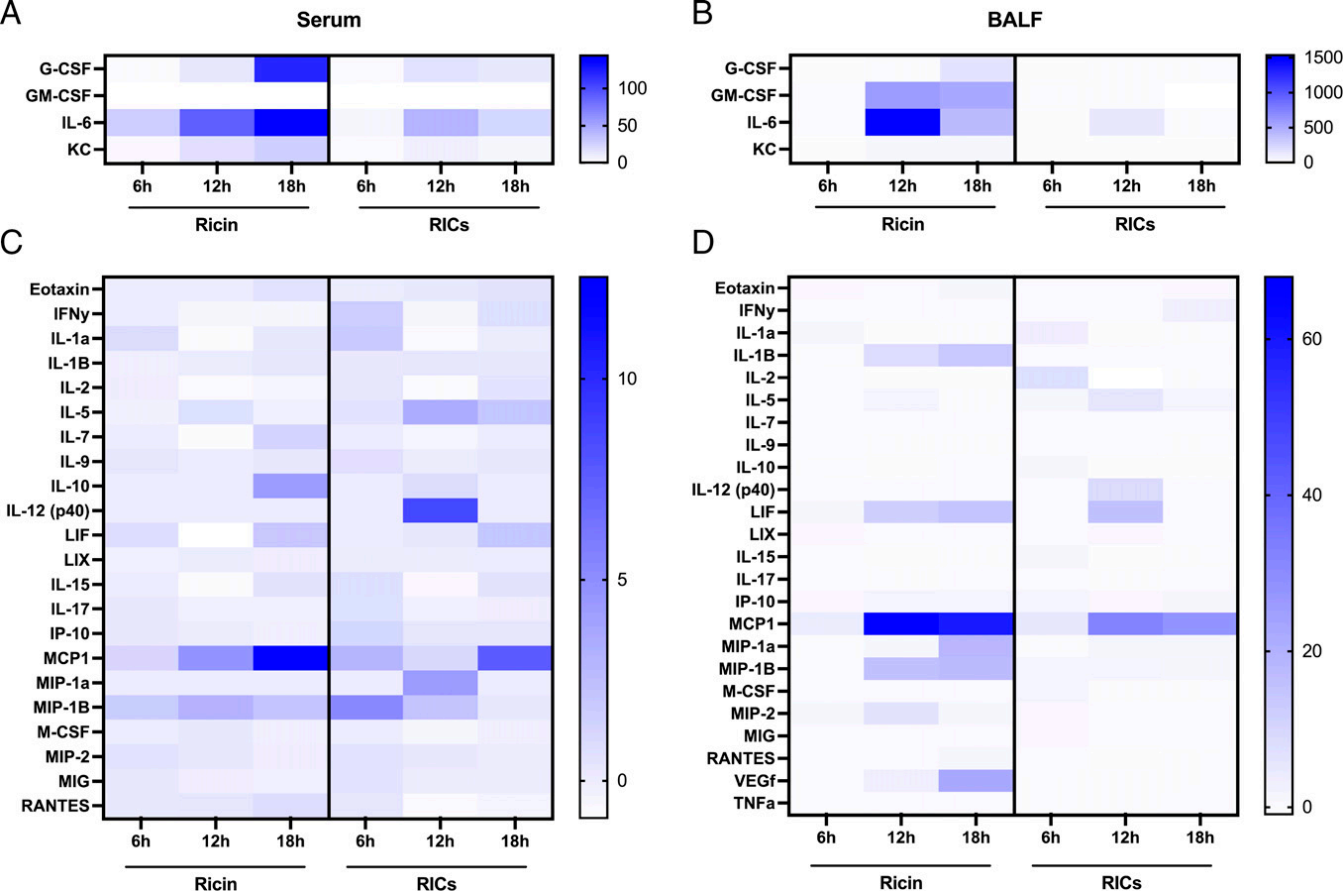
RICs induce an intermediate inflammatory environment within the respiratory tract. RIC, RT, or PBS was administered to mice by the i.n. route before harvest of serum and BALF at 6, 12, or 18 h post-treatment for cytokine content analysis. For each treatment at each time point, *n* = 6. Concentrations are expressed as fold change over PBS-treated samples. Notable inflammatory cytokines produced in high quantities in both (**A**) serum and (**B**) BALF. Cytokines with a nonzero fold change at any tested time point are shown in (**C**) for serum samples and (**D**) for BALF samples. Scales for fold change heatmaps in (A)–(D) vary. Mean group concentration values for all samples, including those with no change, and their statistical significance relative to control samples are presented in Supplemental Table I. Special attention should be given to the varying scales associated with the heatmaps. Significance of concentrations and fold change for each cytokine and sample type among RT- and RIC-treated mice were determined by unpaired, two-tailed Welch’s *t* tests, using cytokine concentrations of PBS-treated animals as baseline values.

We next sought to investigate the impact of RIC exposure on local inflammatory cytokine and chemokine levels, reasoning that the nature of the response may provide a clue to the underlying factors that drive durable anti-toxin B cell responses. To address this question, we collected serum and BALF from mice 6, 12, and 18 h after i.n. RIC (20 µg PB10, 20 µg SylH3, 1 µg RT) administration and then subjected samples to a 32-plex mouse cytokine/chemokine array. We also collected samples from groups of mice treated i.n. with just RT (1 µg), the PB10/SylH3 mAb mixture (20 µg each mAb) alone, or vehicle (PBS). The data presented in this study represent the combined average concentrations and fold changes of two independent replicates. In each replicate, there were three mice per group. Therefore, the averages presented in Fig. 2 and Supplemental Table I constitute six animals per treatment at any given time point.

Although PBS did not induce any notable changes in inflammatory cytokines in either serum or BALF, the PB10/SylH3 mAb mixture did transiently stimulate a variety of cytokines within the panel, although the changes were not statistically significant (Supplemental Table I). BALF samples were more strongly impacted than serum samples, suggesting changes may be associated with local FcR activation.

On the other hand, i.n. delivery of RT stimulated the secretion of a variety of proinflammatory cytokines and chemokines in both serum and BALF to levels that were orders of magnitude higher than background, including G-CSF, IL-6, KC (CXCL1), MCP-1, and MIP-1β (Supplemental Table I). Those same analytes (except KC) were detected in serum of nonhuman primates exposed to RT (8). In the mice, upticks in cytokine and chemokine levels following RT exposure were generally evident in the BALF first and then in serum, consistent with the diffusion of toxin and toxin-induced inflammation from the lung mucosa (Fig. 2). IL-6 stood out because its levels increased >1500-fold in BALF as compared with control animals. IL-6 has previously been implicated as a driver of RT-induced lung pathophysiology in both murine and nonhuman primate models of aerosolized RT exposure (8, 11, 23). Intranasal delivery of RICs stimulated a largely muted inflammatory response in the BALF and serum, as compared with RT itself, apart from KC and IL-6 (Fig. 2; Supplemental Table I). For example, IL-6 levels increased 135-fold in BALF at 12 h following RIC exposure without a corresponding increase in serum IL-6 levels. Collectively, these results suggest that inflammatory responses following RICs are not only are dampened relative to RT but are confined to the lung environment.

Elevated levels IL-6 in the BALF following RIC exposure could contribute to the observed onset of RT-specific IgG by multiple mechanisms, including B cell and T follicular helper cell differentiation (24). To address the role of IL-6 in our model, BALB/c mice were treated with an IL-6–neutralizing mAb (clone MP5-20F3, Bio X Cell) prior to and following RIC immunization. The IL-6–neutralizing mAb regimen resulted in undetectable levels of IL-6 in serum and a significant, albeit partial, reduction in IL-6 levels in BALF (Supplemental Fig. 1A, 1B). However, the IL-6–neutralizing mAb treatment had no impact on the kinetics or magnitude of RT-specific serum IgG levels following RIC treatment as compared with control mice (Supplemental Fig. 1C). Although these results suggest that IL-6 may not be necessary for the onset of RT-specific Abs following RIC exposure, we cannot exclude the possibility that the reduced amounts of IL-6 detected in the BALF are sufficient to promote toxin-specific Ab responses. Further studies aimed at complete ablation of IL-6 in the context of RIC exposure will be needed to resolve the role of this cytokine in inducing downstream RT-specific Ab responses.

## Discussion

Abs in mucosal secretions of the upper and lower airways play a central role in immunity and tissue homeostasis by preventing the uptake of allergens, toxins, and pathogenic agents (25). Indeed, there are multiple mechanisms within the airways to purge Ag-Ab complexes, including mucociliary clearance and phagocytosis by alveolar macrophages. However, there is evidence to suggest that Ab-Ag complexes may also be sampled by APCs within the lung and/or draining lymph nodes with consequences for both mucosal and systemic immunity. Specifically, we reported that, in mice, i.n. delivery of RICs elicits the rapid onset of high-titer, toxin-neutralizing IgG Abs that persist for months (17). We have now extended those original observations and demonstrated that such responses are T cell dependent and occur in the context of elevated levels of IL-6 and possibly other proinflammatory cytokines and chemokines. Although many questions remain regarding the pathways that result in the onset of toxin-specific B cells, the elevated levels of proinflammatory cytokines/chemokine in BALFs following RIC exposure are consistent with the local sampling of ICs.

Prior to this work, the role of T cell help in mediating the humoral response to RICs was unclear. Although the appearance of anti-RT IgG within days following RIC exposure was evidence of B cell class switching and hinted at T cell involvement, the kinetics of the response were more characteristic of a T-independent reaction (17). We now conclude that CD4^+^ T cells are not only involved but essential for mounting a humoral response to RIC vaccination. The necessity of T cell help is particularly interesting, because previous work reported endogenous IgG generation within roughly 7 d of i.n. RIC administration. This rapid timeline for a T-dependent response is unusual, because T cell activation alone can take 24 h, and germinal centers typically do not begin forming until ∼4 d after initial Ag exposure (26) with full formation ∼7–10 d after Ag encounter (27). Thus, i.n. administration of RICs stimulates an extremely fast T-dependent humoral response.

This observation raises the question of where RICs are being presented in the context of the upper and/or lower airways. Canonically, local APCs migrate to the draining lymph node to present Ag to T cells. However, a growing body of literature suggests that Ag presentation may occur within the lung mucosa (28). For example, a recent study implicated interstitial macrophages in the uptake and local presentation of Ag in a murine model of OVA-induced allergic asthma (29). A role for macrophages in RIC sampling would not be surprising, considering that RT itself is efficiently internalized via the mannose receptor and possibly other C-type lectins (4, 30). Macrophages including lung-derived macrophages also secrete an array of proinflammatory cytokines, including IL-6, in response to RT (6, 7). The same could hold true for RICs, because we have shown that PB10 and SylH3 do not necessarily preclude RT’s ability to adhere to lung macrophages (11). Whether macrophages discriminate between RT and RICs is an interesting question, especially considering recent studies demonstrating that virus-Ab complexes elicit different degrees of inflammasome activation and inflammatory chemokines/cytokines compared with virus alone (31).

The potential of ICs as platforms for systemic and mucosal vaccination has largely been viewed through the lens of augmenting Ag uptake and presentation by promoting Fc receptor interactions (16). In this respect, RICs are different in that PB10 and SylH3 likely function as a means of attenuating RT rather than enhancing FcγR-mediated uptake. For example, we reported that RICs generated with PB10 and SylH3 LALAPG derivatives, which are unable to engage with FcγRs, were as immunostimulatory as PB10 and SylH3 IgG1 (17). Similarly, RIC immunostimulatory activity was unaffected in FcγR-deficient mice. The fact that the proinflammatory cytokine/chemokine profile of RICs resembles a damped-down version of RT is also consistent with PB10 and SylH3 serving not to redirect RT specific APCs but to attenuate RT such that it retains its intrinsic antigenicity without cytotoxicity. As appealing a model as that is, it is not the whole story, because the kinetics of de novo RT Ab onset following RIC exposure are distinct from those elicited by low-dose RT (17). Sorting the intrinsic and extrinsic factors that underlie the mucosal immunogenicity of RICs has potential application to mucosal adjuvants and vaccines.

## Supplementary Material

Supplemental Table 1 (PDF)
