# Supplementary material for: Inflammatory Profiles Induced by Intranasal Immunization with Ricin Toxin-immune Complexes
Source: Immunohorizons. 2024 Jun 26;8(6):457–63. doi: 10.4049/immunohorizons.2400007 (PMC11220739; doi:10.4049/immunohorizons.2400007)
Supplement: Supplemental Table 1 (PDF) [file IH_2400007_Supplemental_1.pdf]

Supplemental Figure 1. Role of IL-6 in antibody onset following i.n. RICs vaccination

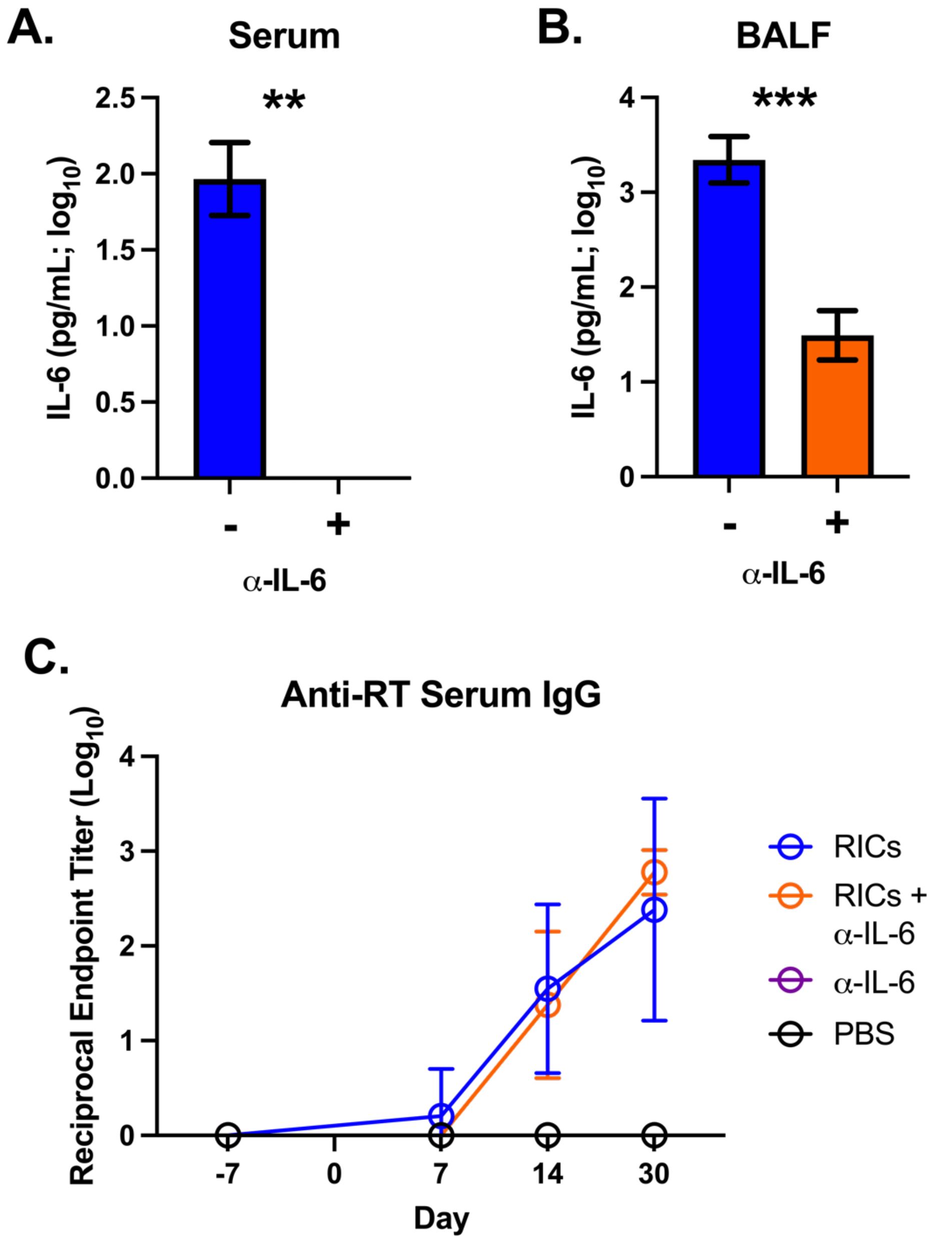

Supplemental Table I. Cytokine Concentrations and Fold Changes

Cytokine	Treat	Source	pg/mL[a,b]			Fold Change[b,c]		
			6h	12h	18h	6h	12h	18h
G-CSF	RT	Serum	351.87	1738.71*	11032.59**	0.57	13.38	119.03
		BALF	380.10	2251.47*	12954.94**	0.74	17.83	155.14
	RICs	Serum	840.17	7	1154.41	2.75	15.98	11.56
		BALF	833.95	2119.11	1214.11	2.81	16.72	13.63
	mAbs	Serum	1272.48	1227.35	449.77	4.69	9.15	3.89
		BALF	596.48	613.02	181.14	1.72	4.13	1.18
	PBS	Serum	223.81	120.94	91.91	0	0	0
		BALF	218.93	119.56	82.97	0	0	0
Eotaxin	RT	Serum	367.71	312.31	441.24	-0.03	-0.02	0.54
		BALF	310.62	226.71	313.49	0.68	0.30	0.79
	RICs	Serum	390.34	365.19	421.74	0.03	0.14	0.47
		BALF	250.72	219.15	261.64	0.36	0.26	0.50
	mAbs	Serum	398.21	330.09	318.15	0.05	0.03	0.11
		BALF	217.72	121.66	55.11	0.18	-0.30	-0.68
	PBS	Serum	377.55	319.15	286.62	0	0	0
		BALF	184.83	172.80	174.71	0	0	0
GM-CSF	RT	Serum	0	0	0	0	0	0
		BALF	25.87	587.20	518.85	24.87	586.20	517.85
	RICs	Serum	0	0	0	0	0	0
		BALF	14.37	9.56	0	13.37	8.56	0
	mAbs	Serum	0	0	0	0	0	0
		BALF	14.26	9.96	1.91	13.26	8.96	0.91
	PBS	Serum	0	0	0	0	0	0
		BALF	0	0	0	0	0	0
IFNγ	RT	Serum	0	0	0	0	-0.48	-0.56
		BALf	0	0	0	0	0	0
	RICs	Serum	2.71	0	3.93	1.71	-0.48	0.74

		BALF	0	0	3.93	0	0	2.93
	mAbs	Serum	0	0	0	0	-0.48	-0.56
		BALF	0.92	1.18	1.23	-0.08	0.18	0.23
	PBS	Serum	0	1.93	2.27	0	0	0
		BALF	1.00	1.00	1.00	0	0	0
IL-1α	RT	Serum	177.89	134.62	142.30	0.91	-0.78	0.18
		BALF	152.85	108.71	105.99	1.44	-0.82	-0.09
	RICs	Serum	266.73	160.01	126.61	1.86	-0.74	0.05
		BALF	283.35	117.36	81.24	3.53	-0.81	-0.31
	mAbs	Serum	170.01	226.70	150.13	0.82	-0.63	0.24
		BALF	85.08	50.64	26.06	0.36	-0.92	-0.78
	PBS	Serum	93.24	611.23	120.78	0	0	0
		BALF	62.56	609.32	117.10	0	0	0
IL-1β	RT	Serum	0.90	1.09	1.22	-0.13	0.06	0.36
		BALF	0.90	9.06	13.05	-0.10	8.06	13.54
	RICs	Serum	1.32	1.42	1.03	0.28	0.38	0.15
		BALF	1.32	1.42	1.03	0.32	0.42	0.15
	mAbs	Serum	1.29	1.03	1.00	0.26	0	0.11
		BALF	7.99	7.81	6.15	6.99	6.81	5.85
	PBS	Serum	1.03	1.03	0.90	0	0	0
		BALF	1.00	1.00	0.90	0	0	0
IL-2	RT	Serum	0	1.64	1.39	-0.04	-0.68	-0.43
		BALf	0	1.64	1.39	-0.04	-0.86	-0.78
	RICs	Serum	1.43	1.13	3.46	0.37	-0.78	0.42
		BALF	9.07	1.13	3.46	7.72	-0.90	-0.46
	mAbs	Serum	4.42	7.78	3.79	3.25	0.54	0.55
		BALF	7.99	7.81	6.15	6.99	6.81	5.85
	PBS	Serum	1.04	5.05	2.44	0	0	0
		BALF	1.00	1.00	0.90	0	0	0
IL-4	RT	Serum	0	0	0	0	0	0
		BALF	0	0	0	0	0	0

	RICs	Serum	0	0	0	0	0	0
		BALF	0	0	0	0	0	0
	mAbs	Serum	0	0	0	0	0	0
		BALF	5.57	3.54	1.26	4.35	-0.69	-0.80
	PBS	Serum	0	0	0	0	0	0
		BALF	1.04	11.31	6.39	0	0	0
IL-3	RT	Serum	0	0	0	0	0	0
		BALF	0	0	0	0	0	0
	RICs	Serum	0	0	0	0	0	0
		BALF	0	0	0	0	0	0
	mAbs	Serum	0	0	0	0	0	0
		BALF	0.85	0.87	0.70	-0.15	-0.13	-0.30
	PBS	Serum	0	0	0	0	0	0
		BALF	1.00	1.00	1.00	0	0	0
IL-5	RT	Serum	22.39	15.28	4.31	-0.33	0.70	-0.21
		BALF	16.59	16.79	5.82	-0.03	1.67	-0.11
	RICs	Serum	48.34	39.36	16.40	0.44	3.39	1.99
		BALF	33.52	42.45	19.05	0.96	5.75	1.92
	mAbs	Serum	50.44	22.75	9.27	0.50	1.54	0.69
		BALF	9.93	6.85	3.62	-0.42	0.09	-0.45
	PBS	Serum	33.60	8.97	5.49	0	0	0
		BALF	17.08	6.29	6.55	0	0	0
IL-6	RT	Serum	43.37	91.26***	145.53****	27.87	90.26	144.53
		BALF	196.28**	1717.61**	2349.98***	20.50	1536.38	400.67
	RICs	Serum	8.15	43.56	22.99	4.43	42.56	21.99
		BALF	54.04	153.04*	80.56**	4.92	135.98	12.77
	mAbs	Serum	31.56	10.76	10.73	20.01	9.76	9.73
		BALF	466.44	309.53	50.78	50.09	276.05	7.68
	PBS	Serum	1.50	1.00	1.00	0	0	0
		BALF	9.13	1.12	5.85	0	0	0

IL-7	RT	Serum	0	0	2.25	0	-0.88	1.25
		BALF	0	0	0	0	0.20	0
	RICs	Serum	0	4.95	0	0	-0.40	0
		BALF	0	0	0	0	0.20	0
	mAbs	Serum	1.00	1.00	0.84	0	-0.88	-0.16
		BALF	1.00	1.00	1.00	0	0.20	0
	PBS	Serum	1.00	8.29	1.00	0	0	0
		BALF	1.00	0.84	1.00	0	0	0
IL-9	RT	Serum	88.91	79.88	73.64	0.36	0.01	0.19
		BALF	149.27	125.90	161.97	0.16	-0.33	-0.21
	RICs	Serum	105.65	87.66	74.07	0.62	0.11	0.20
		BALF	151.09	185.96	121.28	0.17	-0.01	-0.41
	mAbs	Serum	107.41	75.56	72.80	0.65	-0.04	0.18
		BALF	149.19	164.07	153.60	0.16	-0.12	-0.25
	PBS	Serum	65.21	78.84	61.86	0	0	0
		BALF	129.12	187.28	205.39	0	0	0
IL-10	RT	Serum	0	0	5.23	0.01	0	4.23
		BALF	0	2.02	7.13	-0.17	-0.80	0.38
	RICs	Serum	0	1.88	0	0.01	0.88	0
		BALF	2.26	1.88	0	0.88	-0.81	-0.81
	mAbs	Serum	1.00	1.00	1.00	0.01	0	0
		BALF	1.53	1.18	1.20	0.27	-0.88	-0.77
	PBS	Serum	0.99	1.00	1.00	0	0	0
		BALF	1.20	9.90	5.18	0	0	0
IL-12 (p40)	RT	Serum	0	0	0	0	0	0
		BALF	0	0	0	0	0	0
	RICs	Serum	0	9.62	0	0	8.62	0
		BALF	0	9.62	0	0	8.62	0
	mAbs	Serum	1.00	1.00	1.00	0	0	0
		BALF	1.00	1.00	1.95	0	0	0.95
	PBS	Serum	1.00	1.00	1.00	0	0	0

		BALF	1.00	1.00	1.00	0	0	0
IL-12 (p70)	RT	Serum	0	0	0	0	0	0
		BALF	0	0	0	0	0	0
	RICs	Serum	0	0	0	0	0	0
		BALF	0	0	0	0	0	0
	mAbs	Serum	0	0	0	0	0	0
		BALF	0.29	0.29	0	0.29	0.29	0
	PBS	Serum	0	0	0	0	0	0
		BALF	0	0	0	0	0	0
LIF	RT	Serum	1.85	0	2.57	0.85	-0.93	1.97
		BALF	1.85	12.53	15.59	0.85	11.53	14.59
	RICs	Serum	0	17.46	2.59	0	0.23	2.00
		BALF	0	16.39	1.41	0	15.39	0.41
	mAbs	Serum	0	0	3.32	0	-0.93	2.83
		BALF	0.88	0.56	0.08	0.88	0.56	0.08
	PBS	Serum	0	14.20	0.87	0	0	0
		BALF	0	0	0	0	0	0
IL-13	RT	Serum	0	0	0	0	0	0
		BALF	0	0	0	0	0	0
	RICs	Serum	0	0	0	0	0	0
		BALF	0	0	0	0	0	0
	mAbs	Serum	0	0	0	0	0	0
		BALF	0	10.81	0	0	10.81	0
	PBS	Serum	0	0	0	0	0	0
		BALF	0	0	0	0	0	0
LIX	RT	Serum	3534.98	4913.72	4207.07	-0.22	0.06	-0.05
		BALF	2451.17	1883.90	1512.10	0.51	0.31	-0.05
	RICs	Serum	4872.74	4783.88	4396.08	0.07	0.03	-0.01
		BALF	1778.85	2104.48	1810.58	0.10	0.46	0.14
	mAbs	Serum	5154.43	4844.09	3501.03	0.14	0.04	-0.21
		BALF	660.02	439.03	238.66	-0.59	-0.69	-0.85

	PBS	Serum	4534.86	4656.80	4443.47	0	0	0
		BALF	1618.37	1438.32	1582.52	0	0	0
IL-15	RT	Serum	5.42	10.09	11.61	-0.01	-0.82	0.48
		BALF	4.51	9.72	6.03	0.31	-0.65	-0.13
	RICs	Serum	9.47	20.52	11.41	0.74	-0.63	0.45
		BALF	8.03	6.75	5.74	1.33	-0.76	-0.17
	mAbs	Serum	6.14	11.12	18.02	0.13	-0.80	1.29
		BALF	17.56	18.32	10.66	4.09	-0.35	0.55
	PBS	Serum	5.45	55.06	7.86	0	0	0
		BALF	3.45	27.98	6.90	0	0	0
IL-17	RT	Serum	1.24	0.92	1.44	0.24	-0.25	-0.20
		BALF	1.30	0.88	1.10	0.30	-0.28	0.10
	RICs	Serum	1.66	0.94	1.67	0.66	-0.23	-0.08
		BALF	1.36	0.94	1.28	0.36	-0.23	0.28
	mAbs	Serum	1.28	2.08	1.00	0.28	0.70	-0.45
		BALF	3.97	2.44	0.89	2.97	1.00	-0.11
	PBS	Serum	1.00	1.22	1.81	0	0	0
		BALF	1.00	1.22	1.00	0	0	0
IP-10	RT	Serum	141.89	141.97	116.50	0.24	0.10	-0.15
		BALF	115.87	222.41	233.21	0.49	1.59	1.59
	RICs	Serum	239.76	172.80	190.08	1.10	0.34	0.39
		BALF	202.20	141.53	179.15	1.60	0.65	0.99
	mAbs	Serum	232.01	182.92	124.68	1.03	0.42	-0.09
		BALF	281.46	162.95	69.79	2.63	0.90	-0.23
	PBS	Serum	114.37	128.49	137.06	0	0	0
		BALF	77.63	85.83	90.07	0	0	0
KC (CXCL1)	RT	Serum	135.96	437.44***	369.34***	3.25	16.84	25.63
		BALF	429.28*	1830.09***	1106.68****	4.14	36.96	39.18
	RICs	Serum	92.17	220.42	61.46	1.88	7.99	3.43

		BALF	115.66	312.52**	105.68*	0.39	5.48	2.84
	mAbs	Serum	128.18	54.38	35.87	3.00	1.22	1.59
		BALF	366.36	303.95	136.45	3.39	5.30	3.95
	PBS	Serum	32.01	24.52	13.87	0	0	0
		BALF	83.46	48.21	27.54	0	0	0
MCP1	RT	Serum	22.29	44.80*	41.33**	1.21	4.65	12.53
		BALF	34.36*	68.96**	60.27***	4.14	67.96	59.27
	RICs	Serum	39.58	15.84	26.04*	2.92	1.00	7.52
		BALF	39.18	33.11	28.46	4.86	32.11	27.46
	mAbs	Serum	43.78	11.50	10.00	3.34	0.45	2.27
		BALF	47.21	23.28	3.54	6.06	22.28	2.54
	PBS	Serum	10.09	7.92	3.05	0	0	0
		BALF	6.69	1.00	1.00	0	0	0
MIP-1α	RT	Serum	0	0	0	0	0	0
		BALF	7.24	71.62	97.33	-0.57	0.95	18.07
	RICs	Serum	0	5.34	0	0	4.34	0
		BALF	13.11	26.05	5.10	-0.22	1.30	1.48
	mAbs	Serum	0	0	0	0	0	0
		BALF	284.14	155.39	80.01	15.92	12.71	37.83
	PBS	Serum	0	0	0	0	0	0
		BALF	16.80	11.33	2.06	0	0	0
MIP-1β	RT	Serum	10.54	14.44	17.06	1.72	3.11	2.10
		BALF	10.54	71.62	97.33**	0.32	15.31	16.67
	RICs	Serum	24.10	11.23	6.68	5.22	2.20	0.21
		BALF	24.10	13.38	11.11	2.02	2.05	1.02
	mAbs	Serum	22.98	13.21	7.98	4.93	2.76	0.45
		BALF	2966.96	2071.37	124.55	370.89	470.77	21.61
	PBS	Serum	3.87	3.51	5.51	0	0	0
		BALF	7.98	4.39	5.51	0	0	0
M-CSF	RT	Serum	0	2.83	0	0	0.18	-0.17

		BALF	0	2.83	1.31	0	0.18	0.09
	RICs	Serum	0	0	0	0	-0.58	-0.17
		BALF	2.83	0	0	1.83	-0.58	-0.17
	mAbs	Serum	1.02	1.00	1.88	0.02	-0.58	0.56
		BALF	3.43	5.62	5.19	2.43	1.35	3.33
	PBS	Serum	1.00	2.40	1.20	0	0	0
		BALF	1.00	2.40	1.20	0	0	0
MIP-2	RT	Serum	105.66	91.89	95.14	0.53	0.19	-0.07
		BALF	112.25	522.59	181.66	0.90	6.19	0.95
	RICs	Serum	103.79	98.54	109.74	0.50	0.28	0.08
		BALF	96.15	88.50	107.44	0.62	0.22	0.16
	mAbs	Serum	86.17	118.55	103.06	0.24	0.54	0.01
		BALF	105.85	105.42	56.88	0.79	0.45	-0.39
	PBS	Serum	69.22	77.07	101.86	0	0	0
		BALF	59.18	72.63	92.98	0	0	0
MIG	RT	Serum	837.91	702.99	585.98	0.28	-0.04	-0.28
		BALF	810.74	708.04	845.99	0.35	0.05	0.17
	RICs	Serum	934.12	760.25	832.78	0.43	0.04	0.03
		BALF	873.40	720.39	775.33	0.46	0.06	0.08
	mAbs	Serum	798.43	819.80	1072.62	0.22	0.13	0.32
		BALF	175.15	392.97	303.97	-0.71	-0.42	-0.58
	PBS	Serum	655.32	728.59	810.10	0	0	0
		BALF	599.96	677.42	720.85	0	0	0
RANTES	RT	Serum	4.84	6.25	4.41	0.17	0.38	0.77
		BALF	4.84	6.25	4.41	0.17	0.38	0.77
	RICs	Serum	5.70	1.47	1.53	0.38	-0.67	-0.38
		BALF	5.70	1.47	1.53	0.38	-0.67	-0.38
	mAbs	Serum	4.87	3.74	4.16	0.18	-0.17	0.67
		BALF	36.38	22.98	9.15	7.79	4.09	2.68
	PBS	Serum	4.14	4.52	2.48	0	0	0
		BALF	4.14	4.52	2.48	0	0	0

VEGF	RT	Serum	0	0	0	0	0	0
		BALF	0.73	14.87	52.57	-0.60	3.07	22.43
	RICs	Serum	0	0	0	0	0	0
		BALF	1.60	1.64	1.11	-0.13	-0.55	-0.51
	mAbs	Serum	0	0	0			
		BALF	2.23	3.30	3.46	0.21	-0.10	0.54
	PBS	Serum	0	0	0	0	0	0
		BALF	1.85	3.65	2.24	0	0	0
TNFα	RT	Serum	0	0	0	0	0	0
		BALF	0	2.81	1.31	-0.59	0.23	0.31
	RICs	Serum	0	0	0	0	0	0
		BALF	0	0	0	-0.59	-0.56	0
	mAbs	Serum	0	0	0	0	0	0
		BALF	493.91	140.86	13.27	200.18	60.54	12.27
	PBS	Serum	0	0	0	0	0	0
		BALF	2.46	2.29	1.00	0	0	0

[a], Concentration in MFI determined by Luminex and converted to pg/mL per kit manufacturer instructions; [b], Values are group means (n=6); [c], Fold change relative to mean PBS-treated group concentrations for the respective cytokine, sample source, and time point; *, Significantly different value relative to mean PBS-treated group concentration
